# Digital App Based Cognitive Behaviour Therapy CBT‐I Course Improving Insomnia and Sleep Hygiene: A Randomised Controlled Trial

**DOI:** 10.1111/jsr.70195

**Published:** 2025-10-01

**Authors:** Maren‐Jo Kater, Nina Wegener, Nicolas Morath, Albrecht Vorster

**Affiliations:** ^1^ Developmental Psychology and Developmental Psychopathology, Faculty of Psychology and Sports Science Bielefeld University Bielefeld Germany; ^2^ Independent Psychologist Glücksburg Germany; ^3^ Institute of Sport Science University of Bern Bern Switzerland; ^4^ Swiss Sleep House Bern, Department of Neurology University Hospital Inselspital Bern Bern Switzerland

**Keywords:** bedtime restriction, dCBT‐I, prevention, sleep disorder, smartphone app, stimulus control

## Abstract

Problems with initiating and maintaining sleep are among the most common health complaints, with prevalence rates exceeding 50% depending on the survey. Preventing the progression to chronic insomnia may reduce public healthcare costs and prevent secondary illnesses. This study examined the effectiveness of a novel app‐based digital course using cognitive behavioural therapy for insomnia (CBT‐I) in preventing the manifestation of insomnia among individuals with sub‐threshold to moderate symptoms. Participants were assigned to an intervention group (*N* = 191, ages 20–75) or a waitlist control group (*N* = 72, ages 22–77) and assessed at three time points: Pre‐intervention (T0), Post‐intervention (T1, after 7‐week course) and Follow‐up (T2, 3 months after course initiation) using the Insomnia Severity Index (ISI), Sleep Hygiene Index (SHI) and sleep diary entries. Results showed a significant reduction in insomnia severity (−4.8 ± 3.7 ISI points) and improved sleep hygiene (−3.5 ± 4.5 SHI points) from T0 to T1 (*d* = 1.35, and *d* = 0.69, respectively), with stable effects maintained at the 3‐month follow‐up. Remission was achieved by 48% of the intervention group compared to 18% of the control group. Improvements were also observed in sleep latency, sleep efficiency and reductions in nocturnal awakenings and wake time after sleep onset (*d* = 0.25–0.71). Activating evening activates, napping, irregular bedtimes, uncomfortable sleep environment, perceived stress and rumination significantly reduced (*d* = 0.16–0.59). Notably, 68% of users reported sustained improvements in their sleep. The findings indicate that various sleep hygiene behaviours can be effectively modified through an app‐based CBT‐I intervention.

**Trial Registration:** This study was pre‐registered at Open Science Framework https://osf.io/yj2va

## 1 Introduction

A substantial proportion of the population suffers from difficulties initiating and maintaining sleep. According to recent surveys, approximately 43% of adults in Germany reported experiencing sleep disturbances within the past 12 months (Bocksch 2023). Among these individuals, an estimated 5%–10% develop chronic insomnia (Heidbreder et al. 2025), which is characterised by persistent difficulties with sleep initiation and/or maintenance occurring at least three times per week, accompanied by daytime functional impairment. In the absence of treatment, chronic insomnia tends to persist, with longitudinal studies showing that 70% of patients continue to experience symptoms even 3 years later (Ohayon 2009). Chronic sleep disturbances not only significantly impair quality of life but are also associated with an increased risk of comorbid conditions such as depression, anxiety disorders, cardiovascular disease, diabetes and dementia (Fan et al. 2020; Hertenstein et al. 2023; Shi et al. 2018; von Schantz et al. 2021). The resulting burden on the healthcare system is considerable, as insomnia often leads to absenteeism and increased utilisation of medical services (Bassetti and Welter 2025). In Germany, the 2017 DAK Health Report estimated that 9.4% of employees were affected by insomnia, with approximately four sick days per 100 insured individuals attributed to sleep disorders (Marschall et al. 2017).

Cognitive Behavioural Therapy for Insomnia (CBT‐I) is considered the first‐line, evidence‐based treatment for insomnia (Riemann et al. 2023). Randomised controlled trials have demonstrated the effectiveness of various CBT‐I components in improving sleep quality and reducing comorbid symptoms such as daytime fatigue and psychological distress (Steinmetz et al. 2024). Treating chronic insomnia using CBT‐I is at least as effective as pharmacotherapy, but offers longer‐lasting effects, lower cost and additional improvements in emotional well‐being and quality of life (Alimoradi et al. 2022; Natsky et al. 2020). Nevertheless, access to qualified treatment remains limited for many individuals, driving the development and implementation of digital interventions. In recent years, several digital programs and apps have been developed to deliver CBT‐I content, with multiple randomised controlled trials supporting their efficacy (Simon et al. 2023; Seyffert et al. 2016). The digital format enables low‐threshold, flexible and scalable access to care, which is particularly valuable in underserved areas or where waiting times for therapy are long. While the efficacy of dCBT‐I has been well established in clinical samples, few studies have specifically addressed its preventive use among individuals with sub‐threshold insomnia. The present study therefore evaluates the potential of dCBT‐I to prevent progression from subthreshold to chronic insomnia, thereby addressing an important gap in the literature.

Therefore, the present study aimed to evaluate the efficacy and effectiveness of a preventive online course for the reduction of sleep initiation and maintenance difficulties that has been recently certified by the German Central Prevention Testing Authority of the statutory health insurance funds (Zentrale Prüfstelle Prävention, ZPP). The course was evaluated in a controlled study to explore its potential to prevent the progression of insomnia symptoms to chronic stages in a subclinical sample.

## 2 Methods

### 2.1 Procedure

**FIGURE 1:**
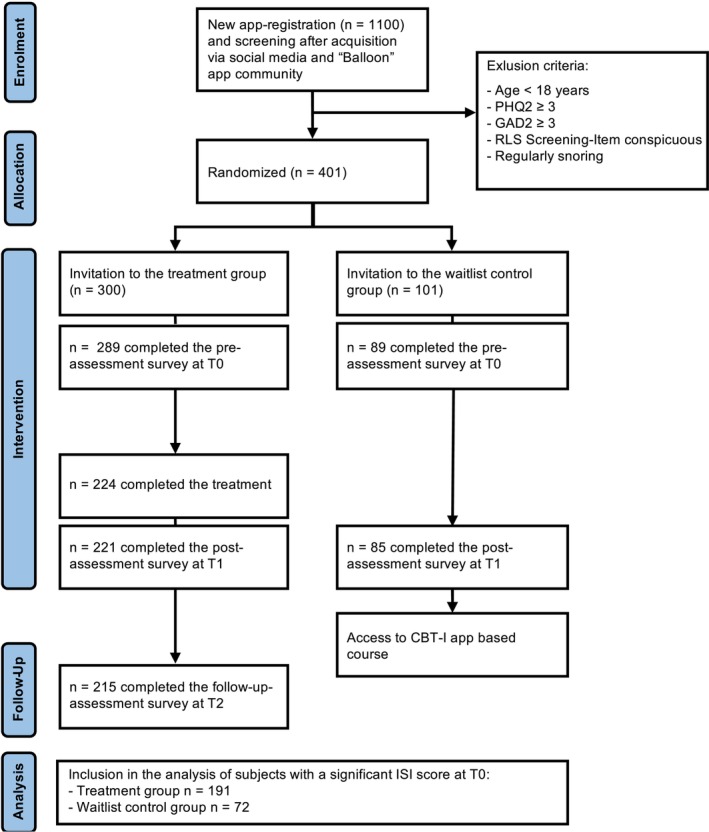
Flowchart of the participant and study outline.

An RCT with two parallel arms (a treatment group and a waitlist control group) was conducted between October 2024 and June 2025 in Germany (Figure 1). Participants were randomised from October 18th to December 10th 2024. After completion of screening questionnaires (described in more detail in the section ‘participants’), participants were randomised using blocked randomisation to one of the two groups: first treatment group was filled (*N* = 265), then control group (*N* = 70), then treatment group (*N* = 25), then control group (*N* = 10), then treatment group (*N* = 10), then control group (*N* = 21). Randomisation was conducted by an independent person who was not involved in study design or data analysis and who had no knowledge of the age or sex of the participants. Randomisation was completed prior to recording of the pre‐test variables (T0). Therefore, there was no knowledge of Insomnia Severity, Sleep Hygiene or Sleep Diary data. Outcome assessors were blinded to group allocation. Participants and clinicians could not be blinded due to the nature of the intervention. The treatment group underwent three measurement points: pre‐intervention (T0), post‐intervention (T1, after completing the 7‐week course) and follow‐up (T2, approximately 12 weeks after course initiation). The treatment group was instructed to complete the app‐based CBT‐I sleep course between T0 and T1 within 42–100 days. Participants who did not complete the program in the given time were not included in the analysis (one participant who completed the course prematurely within 39 days was nevertheless included in the analysis). The waitlist control group underwent T0 and T1 before they received access to the digital sleep course. All participants were instructed to fill out the surveys within 10 days after an e‐mail invitation (five participants who responded within 13 days were still included in the analysis). For complete participation, participants were compensated with a voucher for 6 months of dCBT‐I app access and the opportunity to enter a prize draw for €50.

Sample size was determined using a power analysis conducted with G*Power (Faul et al. 2009). Based on Zachariae et al. (2016), an expected effect size of *f* = 0.5 for treatment effects was assumed. With a statistical power of 80% and a significance level of *p* < 0.05, a minimum sample size of *N* = 128 (64 participants per group) was calculated. In order to ensure adequate statistical power to even detect smaller effects within the preventive intervention group, a larger proportion of participants was randomised to the treatment group compared to the waitlist control. The study protocol was approved by the Hamburg Medical Association Ethics Committee (456403‐nM‐ff) and the trial registered at Open Science Framework https://osf.io/yj2va.

### 2.2 Intervention

The treatment consisted of a digital prevention CBT‐I course ‘Finding restful sleep—an app‐supported online course’ (a course of the ‘7Schläfer’ app; https://7schlaefer.app), composed of seven modules displayed in Table 1, focusing on psychoeducation, sleep hygiene, cognitive techniques, bedtime restriction, behavioural strategies and relaxation techniques according to existing CBT‐I manuals (Müller and Paterok 2010; Backhaus et al. 2011). The program was intended to be completed over the course of 7 weeks chronologically, with one module per week. Modules included psycho‐educative content, quizzes, tasks, a sleep diary and self‐reflection exercises.

**TABLE 1:** Modular structure of the contents of the digital CBT‐I course tested.

Course week	Title	Content	Duration
Week 1	Introduction	Psychoeducation: Sleep architecture, coping with nocturnal awakenings Sleep duration Chronotypes Sleep changes across the lifespan Introduction to the sleep diary	49 min
Week 2	Sleep‐promoting behaviour in the evening and at night	Sleep hygiene Temperature Noise and light Sleep‐promoting environmental factors Social media and smartphone use Alarm clock use Co‐sleeping Regular sleep times Stimulus control	62 min
Week 3	Sleep‐promoting behaviour during the day	Sleep hygiene Daytime napping behaviour Physical activity Caffeine, nicotine and alcohol Sleep promoting diatary behaviour	50 min
Week 4	Evening routine	Psychoeducation Circadian rhythm and melatonin Effects of shift work Evening activities TV use and stimulus control Planning an individual evening routine	43 min
Week 5	Cognitive restructuring	Cognitive restructuring Cognitive relief Paradoxical intention Coping with rumination and catastrophic thinking Thought‐stopping Relaxation techniques Psychoeducation Three elements for falling asleep (sleep pressure, circadian rhythm, relaxation)	38 min
Week 6	Bed time restriction	Psychoeducation Coping with tiredness Morning routines Effects of sleep on health Regularity Cognitive restructuring Dealing with bad nights Introduction to bed time restriction—defining a sleep window	48 min
Week 7	Sleeping better in the long‐term	Psychoeducation Model for the development of sleep disorders Sleep‐related breathing disorders, bruxism and nightmares Sleep medication Coping with relapses Progressive muscle relaxation	78 min

### 2.3 Participants

German speaking participants were recruited via social media posts, in the Balloon‐app (MissionMe, Germany) community and through new registrations in the ‘7Schläfer’ App. Individuals who expressed interest in participating received an e‐mail containing study information, an informed consent form and a screening questionnaire consisting of self‐report sleep measures. Inclusion criteria including being at least 18 years of age, having sufficient German language proficiency, and providing informed consent were applied at study allocation and an Insomnia Severity Index (ISI; Bastien et al. 2001) score ≥ 8 for analysis inclusion. Exclusion criteria were severe mental illness requiring treatment, increased depression and anxiety levels (PHQ‐2 ≥ 3; GAD‐2 ≥ 3), sleep disorders requiring treatment, regular snoring or symptoms of restless legs syndrome. Individuals who had previously registered in the app were excluded if they had listened to more than two units within the app. Of the 401 individuals originally invited, 378 completed the pre‐assessment. Of these, *N* = 224 individuals (77.5%) completed the sleep course, resulting in a drop‐out rate of approximately 22.5%.

Ultimately, a total of *N* = 263 participants were included in the final evaluation (*N* = 191 participants in the intervention group and *N* = 72 in the waitlist control group). All of them completed all measurement points and had at least sub‐threshold insomnia at baseline (T0), indicated by an ISI score ≥ 8. This sample consisted of 215 women, 47 men and 1 diverse person. The average age was 46.95 years (SD = 11.10; age range: 20–77 years, Table 2). The initial average insomnia symptoms measured via the ISI score at T0 were comparable between the treatment group and the waitlist control group (11.59 ± 3.80 and 12.97 ± 3.17), indicating, on average a level of sub‐threshold insomnia in both groups. Sleep Hygiene measured via the SHI was comparable between the treatment and waitlist control group (30.05 ± 5.07 and 29.05 ± 5.69), indicating overall an average level of sleep hygiene in both groups (average SHI = 27–34; Mastin et al. 2006). Pre‐assessment group comparisons between the treatment and the waitlist control group were non‐significant for ISI (*t*(134.7) = −1.11, *p* = 0.27), SHI (*t*(116.6) = −0.65, *p* = 0.52), age (*t*(118.6) = 1.44, *p* = 0.15) and gender (*χ* ^2^(1) = 0.01, *p* = 0.93). The sleep diary showed an average SOL of 28.90 ± 33.84, WASO of 24.90 ± 37.93 and TST of 418.01 ± 85.38.

**TABLE 2:** Demographic characteristics: Age, gender (*n*, %).

*N*	Treatment group	Waitlist control group
	191	72
Age (*M* ± SD)	46.32 ± 10.80	48.62 ± 11.79
Age range	20–75	22–77
18–34 years	28 (14.66%)	11 (15.28%)
35–49 years	89 (46.60%)	28 (38.89%)
50–64 years	69 (36.13%)	28 (38.89%)
> 65 years	5 (2.62%)	5 (6.94%)
Male	35 (18.32%)	12 (16.67%)
Female	156 (81.68%)	59 (81.94%)

Abbreviations: *M* = mean, SD = standard deviation.

### 2.4 Outcome Measures

Primary outcomes included insomnia severity and sleep hygiene behaviour. Insomnia severity was assessed using the Insomnia Severity Index (ISI; Bastien et al. 2001), which assessed perceived sleep difficulties over the past 2 weeks. The seven items are rated on a five‐point Likert scale (0 = not at all, 4 = very severe problem). Higher sum scores, ranging from 0 to 28, indicate a higher degree of symptoms and perceived impact. An ISI score of 0–7 is considered normal, 8–14 as sub‐threshold insomnia, 15–21 as moderate insomnia and values above 21 as severe clinical insomnia (Bastien et al. 2001). In the German version, the ISI showed good internal consistency and a satisfactory test–retest reliability (*α* = 0.83; *r* = 0.78; Dieck et al. 2018).

Sleep hygiene was evaluated using the Sleep Hygiene Index (SHI, Mastin et al. 2006), including total score and item‐level analyses. Participants are asked to rate the frequency to which they have engaged in specific practices and behaviours related to sleep hygiene at 13 items from *never* (1), *rarely* (2), *sometimes* (3), *frequently* (4), to *always* (5). A higher total sum score (range 13–65) is indicative of poor sleep hygiene. The original scale has demonstrated a Cronbach's alpha of 0.66 and good test–retest reliability of *r* = 0.71. Both measures provided via app showed a Cronbach's alpha in this sample of 0.59 (ISI) and 0.65 (SHI).

In the treatment group, a sleep diary offered in the app was used to capture self‐reported sleep measures continuously. The following main parameters were examined: Time in bed (duration between ‘Went to bed’ and ‘Got up’; TIB), sleep onset latency (duration between ‘Went to bed’ and ‘Fallen asleep’; SOL), wake after sleep onset (total duration of awaking periods; WASO), number of awakenings during the night (No. awakenings), sleep efficiency (SE, [TST/TIB * 100]) and sleep quality (‘my sleep quality’ rated on a 5‐point Likert scale from 1 = very poor to 5 = very good; SQ).

At the follow‐up assessment, participants also rated the perceived usefulness of the course (*Usefulness* ‘The course has helped me to sleep better in the long term’), further mental engagement with the course content (*Continued mental engagement* ‘The content of the course has been with me for the last three months’.) and prolonged use of the sleep diary (*Continued Sleep Documentation* ‘I continue to document my sleep’). All three items were answered on a 7‐point Likert scale ranging from (0) *does not apply at all* to (6) *fully applies*. The duration of course completion was estimated as the time elapsed between T0 and T1, and sleep adherence to sleep documentation was measured via the total number of sleep diary entries recorded.

### 2.5 Statistical Analysis

Data analyses were conducted using R (version 4.5.0). Baseline group differences regarding demographic and clinical characteristics were analysed using independent *t*‐tests (two‐sided) and Pearson *χ* ^2^‐tests. Paired‐sample *t*‐tests were used to assess pre‐post changes from T0 to T1 for the total sample, and separately for gender (male, female) and ISI‐Categories (sub‐threshold insomnia ISI = 8–14, moderate insomnia ISI = 15–21). Note that in this sample no participants had an ISI ≥ 22. Effect sizes were calculated using Cohen's *d*. To evaluate treatment effect on primary outcome ISI‐score, secondary outcome SHI‐score and SHI‐items, separate linear mixed‐effects analyses (using Restricted Maximum Likelihood as the estimator) were calculated. Treatment group (treatment vs. waitlist control), time (T0 vs. T1) and the interaction of treatment × time were entered as fixed factors along with age and gender as control variables, and subject as a random factor. Bootstrap confidence intervals were computed for mixed‐effects models, and significant interaction effects were further explored via post hoc contrasts.

In order to determine the sleep diary parameters, as well as further secondary outcome measures, all sleep variables were automatically checked and adjusted beforehand: A value range was defined for each variable, which limits the probability of false data defined as follows: TST_Max_ and TIB_Max_ = 720 min; SE: 0%–100%, SL_Max_ = 300 min and WASO_Max_ = 360 min. Afterwards, for each of the three assessments, existing daily sleep diary data were averaged to a 2‐week mean. Changes pre‐ to post‐treatment in sleep diary data were explored within the treatment group by using dependent *t*‐tests (two‐sided) and effect sizes (Cohen's *d*).

In a secondary analysis, to evaluate the perceived usefulness, adherence indicators and symptom change with the needed program period, bivariate correlations (Pearson's *r*) were calculated.

## 3 Results

### 3.1 Treatment Effects

#### 3.1.1 Insomnia Severity

**FIGURE 2:**
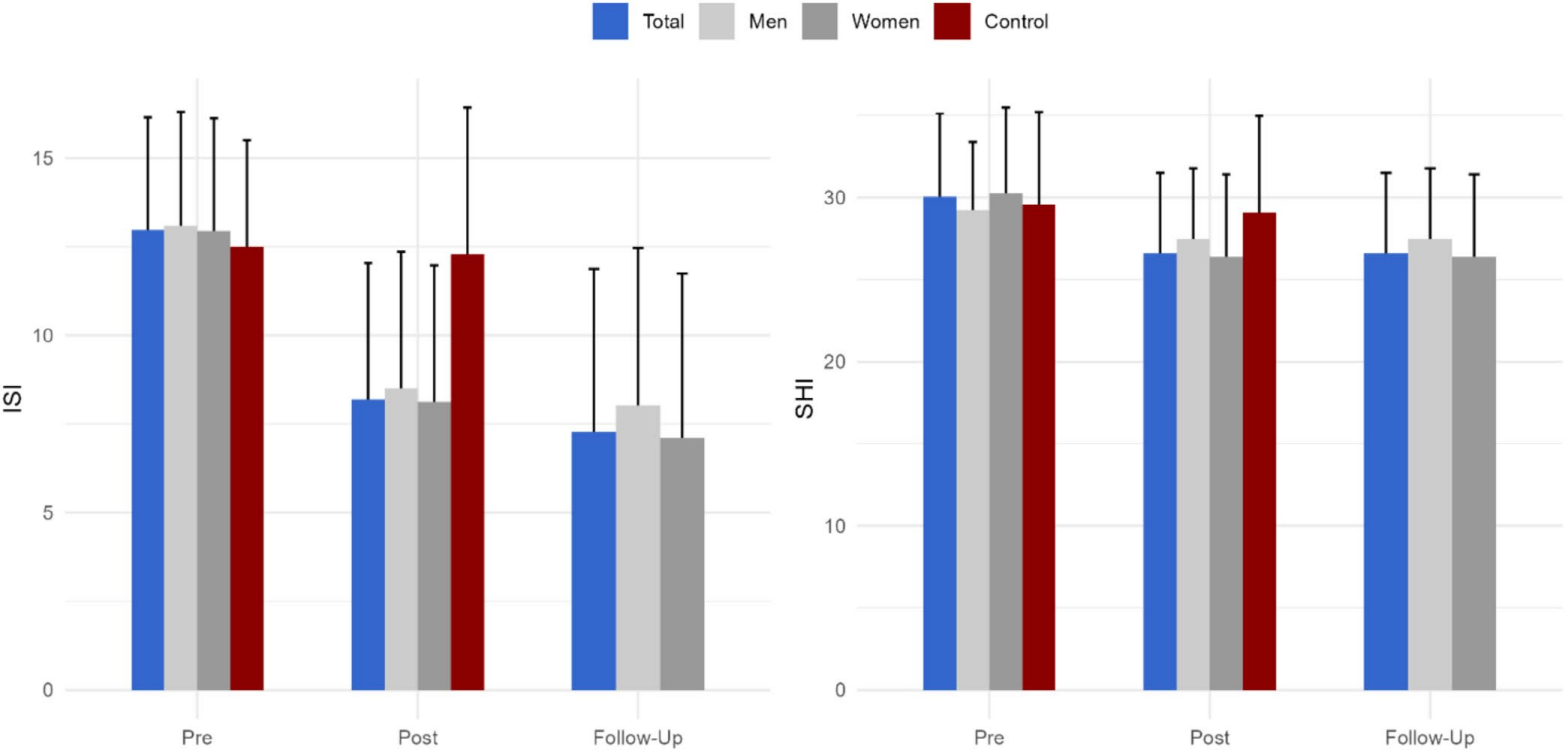
Insomnia severity (ISI) and sleep hygiene (SHI) at pre (T0, before the start of the course), post (T1, after completing the 7‐week course) and follow‐up (T2, 3 months after the start of the digital CBT‐I course). Depicted are the average scores with standard deviations for the ISI and SHI for the treatment group (total and by gender) and the waitlist control group.

Insomnia symptoms measured via the ISI score improved from T0 (12.97 ± 3.17) to T1 (8.18 ± 3.85) for the treatment group (*t*(191) = 17.75, *p* < 0.001, *d* = 1.35, Table 3 and Figure 2). For the main outcome parameter ISI score, a mixed model (*χ* ^2^(3) = 239.1, *p* < 0.001, *R* ^2^ = 0.28) found a significant group × time interaction effect, with post hoc comparisons indicating an improvement from T0 to T1 in the treatment group (*t*(260) = 18.62, *p* < 0.001, *b* = 4.79, SE = 0.26), but no difference in the waitlist control group (*t*(260) = 0.50, *p* = 0.62, *b* = 2.11, SE = 0.42; see Supporting Information for full model). Greatest improvements (6.59 ± 3.92 points) were found for participants with moderate insomnia (*d* = 2.05), while individuals with sub‐threshold insomnia also showed a significant reduction in insomnia symptoms (*d* = 1.44). At post‐treatment, 48% of the treatment group met the criterion for remission (ISI < 8), compared to 18% in the waitlist control group. A Pearson chi‐squared test revealed a significant difference between the two groups on remission (*χ* ^2^(1) = 20.41, *p* < 0.001). At the end of the dCBT‐I program, 21% of participants in the intervention group achieved clinically relevant improvements in insomnia symptoms (defined as a reduction in ISI score of more than 8 points), compared to 0% in the waitlist control group.

**TABLE 3:** Overview of dCBT‐I course outcome for insomnia severity (ISI; *M* ± SD).

ISI	*n*	Pre (T0)	Post (T1)	Follow‐up (T2)	Time effects T0–T1			
					ΔT0–T1	*t*	*p*	Cohen's *d*
Total treatment group	191	12.97 ± 3.17	8.18 ± 3.85	7.27 ± 4.40	−4.79 ± 3.72	17.75	< 0.001	1.35
Female	156	12.94 ± 3.17	8.12 ± 3.85	7.10 ± 4.63	−4.83 ± 3.87	15.60	< 0.001	1.36
Male	35	13.09 ± 3.21	8.49 ± 3.87	8.03 ± 34.44	−4.60 ± 3.05	8.921	< 0.001	1.27
ISI = 8–14	133	11.22 ± 1.79	7.22 ± 3.36	5.97 ± 3.68	−4.00 ± 3.36	13.73	< 0.001	1.44
ISI = 15–21	58	16.98 ± 1.63	10.40 ± 4.00	10.26 ± 5.12	−6.59 ± 3.92	−12.81	< 0.001	2.05

Abbreviations: ISI = Insomnia Severity Index, *M* = mean, *N* = number, SD = standard deviation.

### 3.2 Sleep Hygiene

Paired *t*‐tests showed significant improvements in sleep hygiene measured via the SHI from T0 (30.0 ± 5.07) to T1 (26.60 ± 4.90) for the treatment group (*t*(191) = 10.64, *p* < 0.001, Cohen's *d* = 0.69; Table 4 and Figure 2). For the main outcome parameter SHI, the mixed model (*χ* ^2^(3) = 106.96, *p* < 0.001; *R* ^2^ = 0.12) found a significant group × time interaction effect. Post hoc comparisons indicate an improvement from T0 to T1 in the treatment group (*t*(260) = 11.24, *p* < 0.001, *b* = 3.46, SE = 0.31), but no difference in the waitlist control group (*t*(260) = 0.75, *p* = 0.62, *b* = 0.38, SE = 0.50; see Supporting Information for full model).

**TABLE 4:** Overview of dCBT‐I course outcome for sleep hygiene index (SHI; *M* ± SD).

SHI	*N*	Pre (T0)	Post (T1)	Follow‐up (T2)	Time effects T0–T1			
					ΔT0–T1	*t*	*p*	Cohen's *d*
Total treatment group	191	30.05 ± 5.07	26.60 ± 4.90	25.26 ± 5.09	−3.46 ± 4.49	10.64	< 0.001	0.69
Female	156	30.23 ± 5.25	26.40 ± 5.01	25.06 ± 5.18	3.83 ± 4.61	10.36	< 0.001	0.74
Male	35	29.26 ± 4.13	27.46 ± 4.33	26.14 ± 4.61	1.80 ± 3.47	3.07	< 0.001	0.42
ISI = 8–14	133	30.03 ± 4.67	26.48 ± 4.72	25.05 ± 4.92	−3.55 ± 4.13	9.91	< 0.001	0.76
ISI = 15–21	58	30.10 ± 5.93	26.86 ± 5.33	25.76 ± 5.45	−3.24 ± 5.25	4.70	< 0.001	0.57

Abbreviations: *M* = mean, *N* = number, SD = standard deviation, SHI = Sleep Hygiene Index.

Several sleep hygiene behaviours improved significantly between T0 and T1 in the treatment group, including napping (*d* = 0.23), irregular bed times (*d* = 0.16), feeling stressed at bedtime (*d* = 0.51), rumination in bed (*d* = 0.78), engagement in important work before sleep (*d* = 0.45), engagement in activating activates before sleep (*d* = 0.59), sport within 1 h before sleep (*d* = 0.14), using the bed for other activities than sleep (*d* = 0.47), sleeping in an uncomfortable bed (*d* = 0.29) and room (*d* = 0.28).

There were no significant differences for irregular wake times, prolonged bedtimes in the morning, or alcohol/tobacco/caffeine use 4 h before sleep (all *p* < 0.05).

Item‐level mixed model analyses revealed that while behavioural components like irregular bed times and napping showed non‐significant group × time interactions, cognitive‐arousal‐related behaviours demonstrated moderate effects. Mixed‐effects models indicated reduced feelings of stress at bedtime, less engagement in important work before sleep and decreased rumination in bed in the treatment, but not in the waitlist control group. The SHI item‐level analysis is fully displayed at Table 5. At T0, there were no significant group differences pre‐treatment (see Supporting Information, Table S7).

**TABLE 5:** Overview of dCBT‐I course outcome for the extracted sleep hygiene behaviours at item level of the Sleep Hygiene Index (SHI; *M* ± SD).

SHI items	Pre (T0)	Post (T1)	Follow‐up (T2)	Time effects T0–T1			
				ΔT0–T1	*t*	*p*	Cohen's *d*
Napping	1.29 ± 0.63	1.17 ± 0.39	1.18 ± 0.41	0.13 ± 0.55	3.17	< 0.01	0.23
Alcohol, tobacco, caffeine use 4 h before sleep	2.01 ± 1.02	1.96 ± 1.01	1.74 ± 0.87	0.05 ± 0.55	1.32	0.19	0.05
Doing important work before sleep	2.45 ± 0.92	2.04 ± 0.88	1.95 ± 0.89	0.41 ± 0.88	6.44	< 0.001	0.45
Activating activities before sleep	3.07 ± 1.00	2.48 ± 1.00	2.46 ± 1.02	0.59 ± 0.94	8.74	< 0.001	0.59
Sport 1 h before sleep	1.35 ± 0.62	1.27 ± 0.54	1.20 ± 0.45	0.08 ± 0.54	2.16	0.03	0.14
Bed use for things other than sleeping	2.84 ± 1.24	2.28 ± 1.12	2.22 ± 1.08	0.56 ± 1.07	7.21	< 0.001	0.47
Uncomfortable bed	1.54 ± 0.77	1.34 ± 0.65	1.36 ± 0.70	0.21 ± 0.63	4.59	< 0.001	0.29
Uncomfortable sleep environment	1.78 ± 0.92	1.54 ± 0.82	1.46 ± 0.77	0.24 ± 0.72	4.61	< 0.001	0.28
Feeling stressed or nervous at bedtime	2.54 ± 0.74	2.17 ± 0.70	2.13 ± 0.74	0.37 ± 0.80	6.40	< 0.001	0.51
Irregular bedtimes	2.80 ± 0.84	2.66 ± 0.82	2.45 ± 0.73	0.14 ± 0.91	2.07	< 0.05	0.16
Worrying/rumination in bed	3.39 ± 0.77	2.74 ± 0.89	2.62 ± 0.84	0.65 ± 0.87	10.35	< 0.001	0.78
Irregular waketimes	2.74 ± 0.86	2.66 ± 0.78	2.44 ± 0.74	0.08 ± 0.80	1.36	0.18	0.10
Prolonged morning bedtimes	2.23 ± 1.19	2.28 ± 1.10	2.05 ± 1.03	−0.06 ± 1.09	−0.73	0.46	−0.05

### 3.3 Stability of Treatment Effects

A large within effect size between pre‐assessment (T0) and follow‐up (T2) regarding insomnia severity (*d* = 1.40), and moderate effects for sleep hygiene (*d* = 0.94) in the treatment group were found. There were significant improvements for both ISI and SHI between post (T1) and 3 months follow‐up‐assessment (T2) with small effect sizes (*d*_ISI_ = 0.21, *d*_SHI_ = 0.27). Both results indicate long‐lasting treatment effects (see Supporting Information S1).

### 3.4 Sleep Diary

**FIGURE 3:**
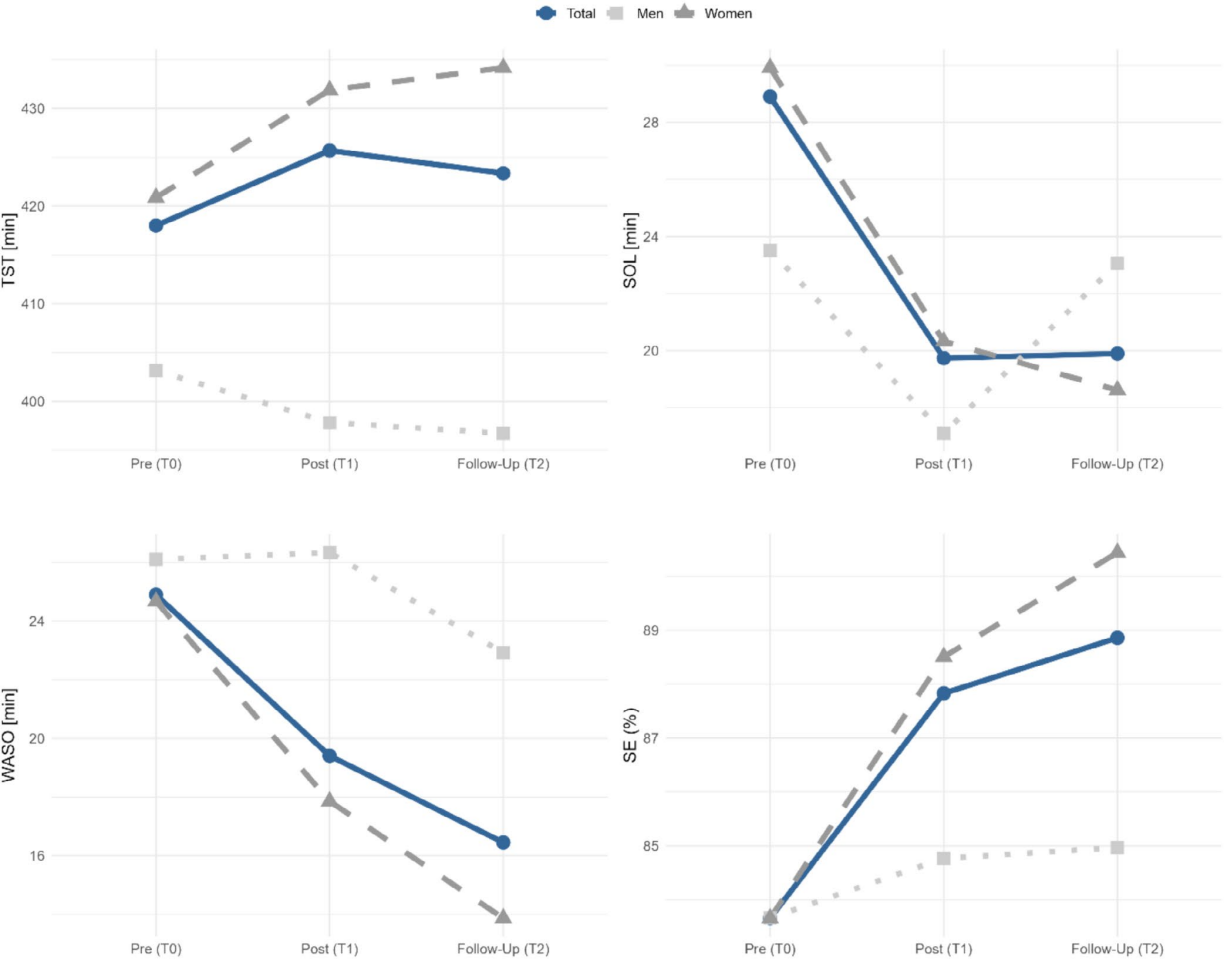
Digital sleep diary parameters at pre‐, post‐ and follow‐up of the application of the dCBT‐I program. Depicted are total sleep time (TST, *N* = 80), sleep onset latency (SOL, *N* = 82), wake after sleep onset (WASO, *N* = 82) and sleep efficiency (SE, *N* = 82). Sample size varies per comparison due to nights without sleep diary entry, and data cleaning. Shown are the average mean scores with standard deviations for the total treatment group and by gender.

For all sleep diary parameters, except total sleep time (TST), significant small to moderate within‐treatment differences between pre‐ (T0) and post‐assessment (T1) were found (*d* = 0.25–0.71; Table 6 and Figure 3). Average sleep onset latency, SOL (−8.40 min, *d* = 0.48), and wake after sleep onset, WASO (−5.96 min, *d* = 0.38), decreased significantly from T0 to T1, as did the number of nocturnal awakenings (−0.25 times, *d* = 0.38). The prolonged total sleep time, TST (+11 min) alongside a shortening of the time in bed, TIB (−11 min, n.s.), led to an increased sleep efficiency, SE (−4.28%, *d* = 0.71). The improvements in sleep parameters correspond to a slight increase in subjective sleep quality (+0.16, range: 1–5, *d* = 0.27).

**TABLE 6:** Overview of dCBT‐I course outcome for sleep diary parameters (*M* ± SD).

Sleep parameter	*N*	Pre (T0)	Post (T1)	Follow‐up (T2)	Time effects T0–T1			
					ΔT0–T1	*t*	*p*	Cohen's *d*
TIB	82	499.10 ± 72.07	481.87 ± 70.42	474.05 ± 63.14	−11.33 ± 40.23	2.55	0.01	0.25
TST	80	418.01 ± 85.38	425.70 ± 79.27	423.36 ± 71.87	11.37 ± 55.55	−1.83	0.07	0.22
SOL	82	28.90 ± 33.84	19.74 ± 20.19	19.90 ± 20.08	−8.40 ± 22.45	3.37	0.001	0.48
WASO	82	24.90 ± 37.93	19.41 ± 32.98	16.45 ± 29.77	−5.96 ± 18.71	2.89	< 0.01	0.38
No. awakenings	83	0.97 ± 1.18	0.74 ± 1.01	0.61 ± 0.85	−0.25 ± 0.61	3.76	< 0.001	0.38
SE	82	83.66 ± 12.68	87.83 ± 9.61	87.86 ± 8.99	4.28 ± 7.38	−5.19	< 0.001	0.71
SQ	80	3.20 ± 1.00	3.39 ± 0.99	3.45 ± 0.95	0.16 ± 0.62	−2.32	0.02	0.27

*Note*: Due to nights without sleep diary entry and data cleaning, the number of sample sizes included varies per comparison (*N*).

The observation of the temporal sleep patterns between T0 and T1 indicates that relatively stable improvements occur for nearly all sleep parameters up to week 8 (see Supporting Information).

### 3.5 Self‐Evaluation of the dCBT‐I Course

Out of the 378 participants that completed the pre‐assessment, *N* = 224 (77.5%) completed the sleep course, resulting in a drop‐out rate of approximately 22.5%. On average, users took 69 ± 16 days to complete the dCBT‐I course (time between T0 and T1), during which they used the integrated sleep diary function of the app, on average, 39 ± 23 times. At follow‐up, 68% of participants reported being confident that the course had sustainably improved their sleep (*n* = 129; i.e., *Usefulness* ≥ *Rather true*). Additionally, 22.5% reported continued mental engagement with the program content (*n* = 43; i.e., *Continued Mental Engagement* ≥ *Rather true*), and 51.8% reported ongoing sleep documentation after completing the program (*n* = 99; i.e., *Continued Sleep Documentation* ≥ *Rather true*).

A correlational analysis was used to investigate the relationship between the self‐reported efficacy of sleep course and adherence (see Table S7). Perceived *Usefulness* significantly correlated with increased *Continued Mental Engagement* (*r* = 0.63, *p* < 0.001) and *Continued Sleep Documentation* (*r* = 0.29, *p* < 0.001) after the end of the course. A higher rating of *Usefulness* is also associated with more frequent use of the sleep diary during the dCBT‐I (*r* = 0.22, *p* < 0.01) and a shorter duration in which the course was completed (*r* = −0.18, *p* < 0.05). In addition, increased *Usefulness was correlated* with a greater reduction in the ISI score both for ΔT0–T1 (*r* = −0.23, *p* < 0.01) and ΔT1–T2 (*r* = −0.33, *p* < 0.001).

## 4 Discussion

The present study evaluated the effectiveness of an app‐based dCBT‐I course to improve sleep among individuals with subclinical to moderate insomnia symptoms, using a randomised controlled trial design. The sample consisted predominantly of participants with subclinical symptom severity, without a formal diagnosis of insomnia. The analysis revealed (1) a reduction in insomnia severity as measured by the Insomnia Severity Index (ISI), (2) an improvement in sleep hygiene as assessed by the Sleep Hygiene Index (SHI) and (3) objective improvements in sleep parameters derived from digital sleep diaries.

### 4.1 Reduction in Insomnia Severity (ISI)

Participation in the dCBT‐I course led to a highly significant reduction in insomnia symptoms, with a mean improvement of −4.79 points on the ISI (Cohen's *d* = 1.35). This effect size is considered large (Cohen 1992) and exceeds the average effects reported in comparable meta‐analyses on digital CBT‐I interventions (e.g., Hedges's *g* = 1.09 in Zachariae et al. 2016; *d* = 0.39 in Soh et al. 2020). Notably, 48.2% of participants in the intervention group (compared to 18% in the control group) achieved remission (ISI < 8), and 21.5% showed a clinically meaningful improvement (≥ 8‐point reduction on the ISI), in contrast to 0% in the control group. The remission rate is comparable to other digital CBT‐I programs (e.g., Ritterband et al. (2022) reported a 40% remission rate, Maurer et al. (2025) a 38% remission rate).

The discrepancy between the relatively high remission rate (48.2%) and the lower proportion of participants showing a clinically meaningful change (21.5%) can be explained by the low baseline ISI scores in the sample, with a mean ISI of 12.97 at study onset—indicative of subclinical insomnia. Thus, many participants did not reach high enough baseline scores to allow for reductions greater than 8 points. These findings underscore the effectiveness of the program, even among individuals with subclinical insomnia symptoms, thereby supporting its potential as a preventive intervention.

At follow‐up, the effect remained stable (T2: *M* = 7.27), indicating sustained impact (*d* = 1.40 from T0 to T2). This long‐term improvement aligns with previous findings demonstrating the durability of CBT‐I effects over several months (Van der Zweerde et al. 2019), and in some cases, up to 10 years post‐intervention (Jernelöv et al. 2022).

### 4.2 Improvements in Sleep Hygiene (SHI) and Sleep‐Related Cognitions

The SHI allowed for a more detailed assessment of sleep behaviour and sleep‐related cognitions targeted by the intervention. Overall, the SHI total score improved significantly from *M* = 30.05 to *M* = 26.60 (*d* = 0.69). These improvements are comparable to Pchelina et al. (2018), who found a reduction from 26.9 ± 7.5 to 23.9 ± 5.7 points pre post the CBT‐I intervention. Significant improvements were found in: (1) ruminative thinking/worry in bed (−0.65 points, *d* = 0.78), (2) perceived stress/nervousness at bedtime (−0.37 points, *d* = 0.51), (3) evening routines/avoidance of evening work activities (−0.41 points, *d* = 0.45), other activating activities (−0.59 points, *d* = 0.59) or sport prior bedtime (−0.08 points, *d* = 0.14), (4) reduction in daytime napping (−0.13 points, *d* = 0.23) and (5) reduction in irregular bedtimes (−0.14 points, *d* = 0.16). These findings suggest the course successfully fostered adaptive sleep‐related behavioural changes and cognitive restructuring, resulting in less stress around sleep and reduced pre‐sleep rumination. Modules targeting rumination control, cognitive restructuring and offloading, evening routines and stimulus control appeared particularly effective—findings that are consistent with prior meta‐analyses (Jansson‐Fröjmark and Norell‐Clarke 2018; Steinmetz et al. 2024).

Moreover, providing modules with advice on creating a sleep‐promoting environment appeared to help participants reorganise their bedroom, resulting in a reduction of discomfort related to the bed (−0.21 points, *d* = 0.29) and the sleeping room (−0.24 points, *d* = 0.28). The quality of sleep is linked to a non‐disturbing environment determined by the factors of light, temperature, noise levels and also the unfavourable mattress or pillow choice (i.e., Caddick et al. 2018; Troynikov et al. 2018). Thus, educating participants about the implications of these disruptive factors could be fundamental for eliminating underlying situational confounders.

No significant changes were observed in items concerning evening alcohol, caffeine, or nicotine consumption—likely due to low baseline levels, as most users reported ‘rare’ use of these substances before bedtime. Similarly, there was no change in prolonged time in bed on weekends, likely reflecting the challenge of implementing consistent wake‐up times—a behaviour that requires considerable self‐discipline despite its proven effectiveness. Potentially, the dCBT‐I course could place greater emphasis on the effective strategy of maintaining regular and restricted bedtimes, which was rather late introduced in week six of the program. However, introducing this component earlier might increase the dropout rate among participants (dropout rates increase after introducing bedtime restriction, see Schuffelen et al. 2023).

### 4.3 Improvements in Sleep Diary Parameters

In addition to self‐report questionnaires, sleep quality was tracked via a continuous digital sleep diary. Significant improvements were observed in nearly all parameters: (1) sleep efficiency (SE) increased from 83.7% to 87.8% (+4.28%, *d* = 0.71), (2) sleep onset latency (SOL) decreased by 8.4 min (*d* = 0.48), (3) wake after sleep onset (WASO) decreased by 5.96 min (*d* = 0.38), (4) the number of nocturnal awakenings declined by 0.25 episodes (*d* = 0.38) and (5) subjective sleep quality improved by 0.16 points (*d* = 0.27). These findings confirm the subjective benefits of the intervention and are consistent with expected outcomes from CBT‐I programs (Hasan et al. 2022; Ye et al. 2016; Ritterband et al. 2022).

The observed decrease in time in bed (TIB) by 11 min, accompanied by an increase in total sleep time (TST) by 11 min, suggests that participants actively implemented the bedtime restriction or sleep compression component of the program. This element is widely regarded as the most potent component within CBT‐I protocols (Steinmetz et al. 2024).

Although the changes in the single sleep parameters appear rather small, there are still meaningful differences in this sample with sub‐threshold or moderate insomnia symptoms as the subjective evaluation of sleep underline.

### 4.4 Perceived Impact and Subjective Evaluation

A total of 68% of participants reported a lasting improvement in their sleep as a result of the course. Perceived sustainability of the effects correlated significantly with reductions in ISI scores (*r* = 0.23), continued cognitive engagement with the course content (*r* = 0.63) and regular use of the sleep diary. These findings highlight the importance of cognitive insight and reflective application as key factors driving success (Ritterband et al. 2022).

### 4.5 Limitations and Future Directions

Several limitations of the study must be considered. Generalisability is limited due to the gender imbalance in the sample (82% female). Moreover, our sample included potentially women undergoing the menopausal transition, a period that can substantially affect sleep quality. However, CBT‐I has been shown to effectively reduce sleep problems in this population (Ntikoudi et al. 2024). Future studies could stratify participants by menopausal status or specifically target this age group to further investigate the effectiveness of CBT‐I across different age, gender and symptom profiles. In addition, the study did not collect systematic information about the use of (sleep) medications or other ongoing health‐related interventions, limiting the attribution of observed effects exclusively to the digital CBT program. Still, subjective evaluation of treatment effects indicates clearly the perceived usefulness as compared to the waitlist control group. Objective measures such as actigraphy could have been employed to complement self‐reports (Kang et al. 2017). The waitlist control group did not have access to the digital sleep diary during the waiting period, as it was embedded within the CBT‐I app and not available as a standalone tool. Therefore, no direct group comparisons can be derived. Moreover, no intention‐to‐treat analysis was conducted, allowing only the interpretation of effect sizes for completers. The lack of detailed adherence metrics for individual course modules limited the identification of specific mechanisms of change. Future studies should include mediation analyses to determine which components (e.g., rumination control vs. sleep restriction) are most effective and relevant for ongoing adherence. Long‐term follow‐up periods of 1–3 years or more would also be valuable.

## 5 Conclusion

The findings of this study demonstrate that the tested dCBT‐I program yields substantial improvements in insomnia symptoms, sleep hygiene behaviours and objective sleep parameters in users with subclinical to moderate symptoms. The integration of cognitive‐behavioural and preventive components appears particularly effective—especially in targeting rumination, sleep‐onset stress and sleep compression. The program thus offers a low‐cost, low‐threshold, scalable intervention with high public health relevance.

## Author Contributions

**Maren‐Jo Kater:** writing – original draft, writing – review and editing, visualization, methodology, formal analysis, software. **Nina Wegener:** conceptualization, investigation, methodology, project administration, writing – review and editing, funding acquisition, data curation. **Nicolas Morath:** data curation, formal analysis. **Albrecht Vorster:** writing – original draft, writing – review and editing, funding acquisition, conceptualization, project administration, supervision, validation.

## Ethics Statement

This study was conducted according to the guidelines of the Declaration of Helsinki. This study was approved by the Hamburg Medical Association Ethics Committee (456403‐nM‐ff).

## Consent

Informed consent was obtained from all participants involved in this study.

## Conflicts of Interest

A.V. developed the concept and content of the 7Schläfer program and receives a share of the company's profits. M.‐J.K. and N.W. received financial compensation for their work. The funder (Gruner + Jahr) had no role in the analyses, or interpretation of data.

## Supporting information


**Data S1:** jsr70195‐sup‐0001‐supinfo.docx.

## Data Availability

The data that support the findings of this study are available from the corresponding author upon reasonable request.
