# Supplementary material for: Digital App Based Cognitive Behaviour Therapy CBT‐I Course Improving Insomnia and Sleep Hygiene: A Randomised Controlled Trial
Source: J Sleep Res. 2025 Oct 1;35(2):e70195. doi: 10.1111/jsr.70195 (PMC13003281; doi:10.1111/jsr.70195)
Supplement: Supplementary file 1 — Data S1: jsr70195‐sup‐0001‐supinfo.docx. [file JSR-35-e70195-s001.docx]

# Supplemental Material

Table S1.

*Comparison for insomnia severity (ISI) and sleep hygiene (SHI) between pre- and follow-up- assessment (T0-T2), and post and follow-up-assessment (T1-T2) for the total treatment group.*

|  | ISI | | | | SHI | | | |
| --- | --- | --- | --- | --- | --- | --- | --- | --- |
|  | *M* ± *SD* | *t* | *p* | Cohen’s *d* | *M* ± *SD* | *t* | *p* | Cohen’s *d* |
| Δ T0-T2 | -5.70 ± 4.14 | 19.01 | < .001 | 1.40 | -4.79 ± 4.71 | 14.07 | < .001 | 0.94 |
| Δ T1-T2 | -0.91 ± 3.46 | 3.64 | < .001 | 0.21 | -1.34 ± 3.54 | 5.21 | < .001 | 0.27 |

Table S2.

*Linear Mixed Model Prediciting ISI-Scores (REML-Estimator)*

| Fixed Effects | b | SE | df | *t* | *p* | 95 %-CI |
| --- | --- | --- | --- | --- | --- | --- |
| Intercept | 12.59 | 1.01 | 281.49 | 12.47 | < .001 | [10.63, 14.39] |
| Group (Control) | 0.50 | 0.50 | 411.78 | 1.01 | .314 | [-0.46, 1.49] |
| Time (T1) | -0.21 | 0.42 | 260.00 | -0.50 | .617 | [-1.06, 0.65] |
| Age | -0.01 | 0.02 | 258.00 | -0.58 | .561 | [-0.04, 0.02] |
| Gender (f) | 0.42 | 0.50 | 258.00 | 0.85 | .394 | [-0.51, 1.45] |
| Treatment × Time | -4.57 | 0.49 | 260.00 | -9.27 | < .001 | [-5.55, -3.67] |
| Random Effects | Var | SD |  |  |  |  |
| ID | 6.308 | 2.512 |  |  |  |  |

*Note*: χ2(3) = 239.1, *p* < .001, R² = 0.28; Reference categories: Group = waitlist control group; time = T0; gender = male.

*Post-hoc contrasts*

| Group | time | b | SE | df | *t* | *p* |
| --- | --- | --- | --- | --- | --- | --- |
| Waitlist control | T0 – T1 | 2.11 | 0.42 | 260 | 0.50 | .62 |
| Treatment | T0 – T1 | 4.79 | .26 | 260 | 18.62 | < .001 |

Table S3.

*Linear Mixed Model Prediciting SHI-Scores (REML-Estimator)*

| Fixed Effects | b | SE | df | *t* | *p* | 95 %-CI |
| --- | --- | --- | --- | --- | --- | --- |
| Intercept | 33.98 | 1.50 | 273.04 | 22.70 | < .001 | [31.00, 36.69] |
| Group (Control) | 0.47 | 0.71 | 362.32 | 0.66 | .501 | [-0.94, 1.86] |
| Time (T1) | -0.38 | 0.50 | 260.00 | -0.75 | .451 | [-1.39, 0.65] |
| Age | -0.10 | 0.03 | 258.00 | -3.81 | < .001 | [-0.14, -0.05] |
| Gender (f) | 0.19 | 0.74 | 258.00 | 0.25 | .802 | [-1.23, 1.71] |
| Treatment × Time | -3.08 | 0.59 | 260.00 | -5.21 | < .001 | [-4.25, -1.99] |
| Random Effects | Var | Sd |  |  |  |  |
| ID | 16.682 | 4.084 |  |  |  |  |

*Note*: χ2(3) = 106.96, *p* <.001, R² = 0.12; Reference categories: Group = waitlist control group; time = T0; gender = male.

*Post-hoc contrasts*

| Group | time | b | SE | df | *t* | *p* |
| --- | --- | --- | --- | --- | --- | --- |
| Waitlist control | T0 – T1 | 0.38 | 0.50 | 260 | 0.75 | .45 |
| Treatment | T0 – T1 | 3.46 | 0.31 | 260 | 11.24 | < .001 |

*Overview of the linear mixed models for the specific sleep hygiene behaviors at SHI item level* - Only models with significant interaction effects reported here. All other models show neither main effects nor interaction effects.

Table S4.

*Linear Mixed Model Prediciting SHI-Item* "*Feeling stressed, nervous at bedtime“* *(REML-Estimator)*

| Fixed Effects | b | SE | df | *t* | *p* | 95 %-CI |
| --- | --- | --- | --- | --- | --- | --- |
| Intercept | 3.42 | 0.20 | 287.87 | 17.08 | < .001 | [3.02, 3.79] |
| Group (Control) | -0.14 | 0.10 | 442.66 | -1.41 | .16 | [-0.33, 0.06] |
| Time (T1) | -0.08 | 0.09 | 260.00 | -0.90 | .37 | [-0.27, 0.11] |
| Age | -0.02 | 0.003 | 258.00 | -4.87 | < .001 | [-0.02, -0.01] |
| Gender (f) | 0.04 | 0.10 | 258.00 | 0.46 | .65 | [-0.15, 0.25] |
| Treatment × Time | -0.29 | 0.11 | 260.00 | -2.62 | .01 | [-0.50, -0.09] |
| Random Effects | Var | Sd |  |  |  |  |
| ID | 0.212 | 0.461 |  |  |  | [0.389, 0.540] |
| Residual | 0.312 | 0.558 |  |  |  | [0.505, 0.609] |

*Note*: χ2(3) = 51.53, *p* <.001, R² = 0.12; Reference categories: Group = waitlist control group; time = T0; gender = male.

*Post-hoc contrasts*

| Group | time | b | SE | df | *t* | *p* |
| --- | --- | --- | --- | --- | --- | --- |
| Waitlist control | T0 – T1 | 0.08 | 0.09 | 260 | 0.90 | .368 |
| Treatment | T0 – T1 | 3.37 | 0.06 | 260 | 6.51 | < .001 |

Table S5.

*Linear Mixed Model Prediciting SHI-Item* "*Doing Important Work Before Bedtime“* *(REML-Estimator)*

| Fixed Effects | b | SE | df | *t* | *p* | 95 %-CI |
| --- | --- | --- | --- | --- | --- | --- |
| Intercept | 2.71 | 0.27 | 276.98 | 10.14 | < .001 | [2.19, 3.19] |
| Group (Control) | 0.21 | 0.13 | 386.45 | 1.61 | .11 | [-0.05, 0.46] |
| Time (T1) | 0.01 | 0.10 | 260.00 | 0.14 | .89 | [-0.19, 0.22] |
| Age | -0.01 | 0.01 | 258.00 | -2.54 | .01 | [-0.02, -0.00] |
| Gender (f) | 0.08 | 0.13 | 258.00 | 0.63 | .53 | [-0.16, 0.35] |
| Treatment × Time | -0.42 | 0.12 | 260.00 | -3.58 | < .001 | [-0.66, -0.21] |
| Random Effects | Var | Sd |  |  |  |  |
| ID | 0.490 | 0.700 |  |  |  | [0.620, 0.792] |
| Residual | 0.360 | 0.600 |  |  |  | [0.543, 0.654] |

*Note*: χ2(3) = 41.20, *p* <.001, R² = 0.05; Reference categories: Group = waitlist control group; time = T0; gender = male.

*Post-hoc contrasts*

| Group | time | b | SE | df | *t* | *p* |
| --- | --- | --- | --- | --- | --- | --- |
| Waitlist control | T0 – T1 | -0.01 | 0.10 | 260 | -0.14 | .889 |
| Treatment | T0 – T1 | 0.41 | 0.06 | 260 | 6.65 | < .001 |

Table S6.

*Linear Mixed Model Prediciting SHI-Item “Worring or Rumination in Bed “* *(REML-Estimator)*

| Fixed Effects | b | SE | df | *t* | *p* | 95 %-CI |
| --- | --- | --- | --- | --- | --- | --- |
| Intercept | 3.84 | 0.23 | 285.14 | 16.51 | < .001 | [3.39, 4.27] |
| Group (Control) | -0.02 | 0.12 | 430.17 | -0.15 | .884 | [-0.24, 0.22] |
| Time (T1) | -0.11 | 0.10 | 260.00 | -1.08 | .280 | [-0.32, 0.10] |
| Age | -0.01 | 0.00 | 258.00 | -3.51 | < .001 | [-0.02, -0.01] |
| Gender (f) | 0.26 | 0.11 | 258.00 | 2.26 | .025 | [0.04, 0.49] |
| Treatment × Time | -0.54 | 0.12 | 260.00 | -4.44 | < .001 | [-0.78, -0.32] |
| Random Effects | Var | Sd |  |  |  |  |
| ID | 0.308 | 0.555 |  |  |  | [0.475, 0.646] |
| Residual | 0.385 | 0.621 |  |  |  | [0.562, 0.677] |

*Note*: χ2(3) = 99.05, *p* <.001, R² = 0.15; Reference categories: Group = waitlist control group; time = T0; gender = male.

*Post-hoc contrasts*

| Group | time | b | SE | df | *t* | *p* |
| --- | --- | --- | --- | --- | --- | --- |
| Waitlist control | T0 – T1 | 0.11 | 0.10 | 260 | 1.08 | .280 |
| Treatment | T0 – T1 | 0.65 | 0.06 | 260 | 10.31 | < .001 |

Table S7.

*Comparisons of sleep hygiene behaviors at item level of the Sleep Hygiene Index (SHI*; *M*±*SD*) in the waitlist control group and treatment group at pre-assessment

|  |  |  | t-test: Treatment vs Control group | | |
| --- | --- | --- | --- | --- | --- |
| SHI Items | Treatment group | Waitlist control group | *t* | *p* | Cohen’s *d* |
| Napping | 1.29 ± 0.63 | 1.38 ± 0.85 | 0.85 | .40 | 0.12 |
| Alcohol, tabacco, caffeine use 4h before sleep | 2.01 ± 1.02 | 2.04 ± 0.94 | .23 | .82 | 0.03 |
| Doing important work before sleep | 2.45 ± 0.92 | 2.24 ± 1.01 | -1.60 | .11 | 0.22 |
| Activating activities before sleep | 3.07 ± 1.00 | 3.04 ± 1.19 | -0.22 | .83 | 0.03 |
| Sport 1h before sleep | 1.35 ± 0.62 | 1.29 ± 0.59 | -0.70 | .49 | 0.19 |
| Bed use for things other than sleeping | 2.84 ± 1.24 | 2.65 ± 1.21 | -1.11 | .27 | 0.15 |
| Uncomfortable bed | 1.54 ± 0.77 | 1.69 ± 0.96 | 0.58 | .56 | 0.08 |
| Uncomfortable sleep environment | 1.78 ± 0.92 | 1.54 ± 0.82 | -0.71 | .48 | 0.10 |
| Feeling stressed or nervous at bedtime | 2.54 ± 0.74 | 2.65 ± 0.65 | 1.09 | .28 | 0.15 |
| Irregular bedtimes | 2.80 ± 0.84 | 2.88 ± 0.85 | 0.63 | .53 | 0.09 |
| Worrying / rumination in bed | 3.39 ± 0.77 | 3.39 ± 0.81 | -0.03 | .97 | 0.00 |
| Irregular waketimes | 2.74 ± 0.86 | 2.63 ± 0.96 | -0.96 | .34 | 0.13 |
| Prolonged morning bedtimes | 2.23 ± 1.19 | 2.07 ± 1.12 | -0.96 | .34 | 0.13 |

**Sleep Diary**

The time courses of sleep between pre- and post-assesment are shown here. The completed sleep diary entries are averaged and displayed for each week. Please note, that the individual duration of program use differs, so that less personal data is available as the number of weeks increases.

Figure S1. Course of the average sleep duration (TST) in minutes for the total group of the treatment group and separately for gender over the time between T0 and T1 per week.
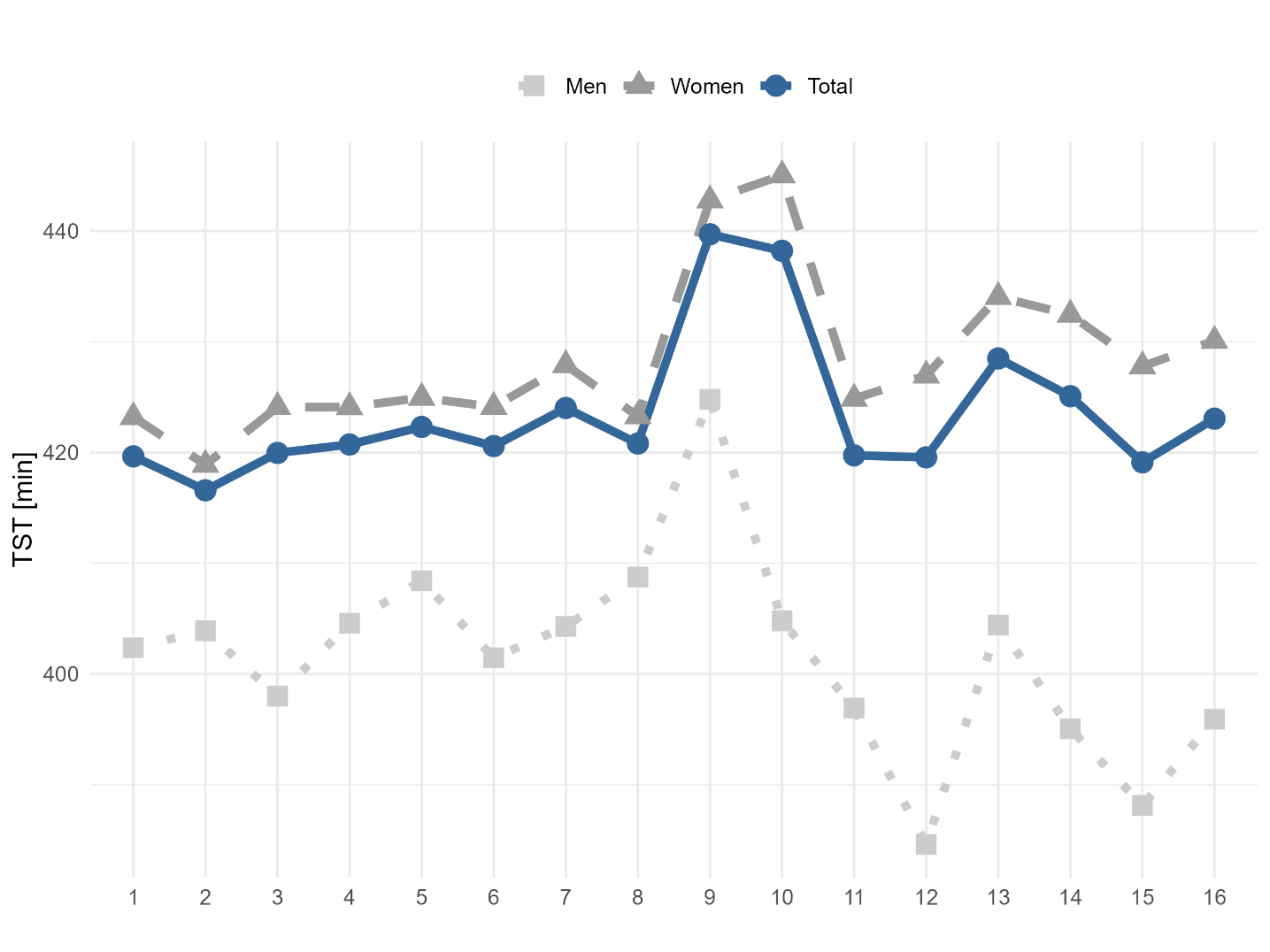


Figure S2. Course of the average sleep onset latency (SOL) in minutes for the total group of the treatment group and separately for gender over the time between T0 and T1 per week.
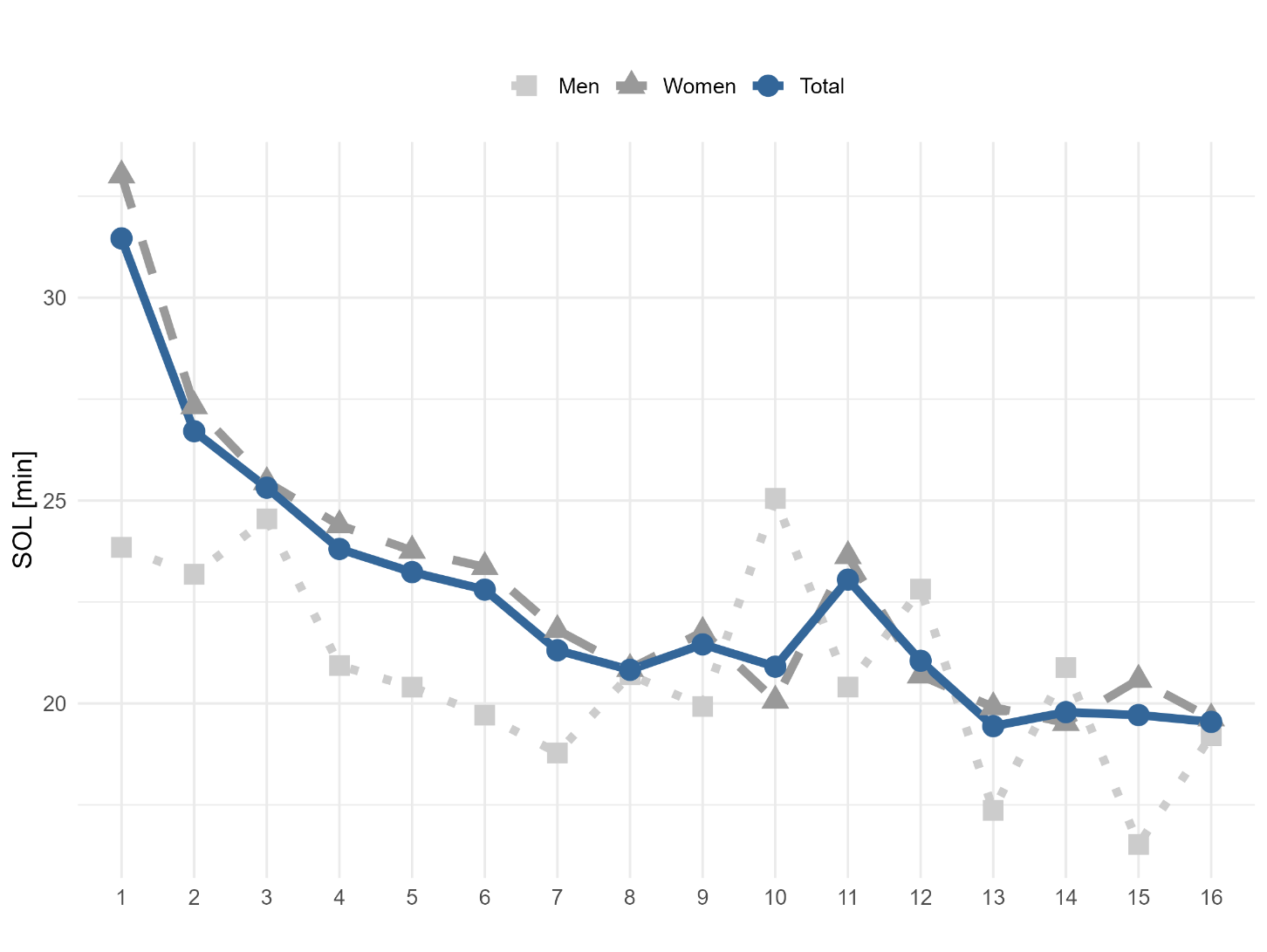


Figure S3. Course of the average wake after sleep onset (WASO) in minutes for the total group of the treatment group and separately for gender over the time between T0 and T1 per week.
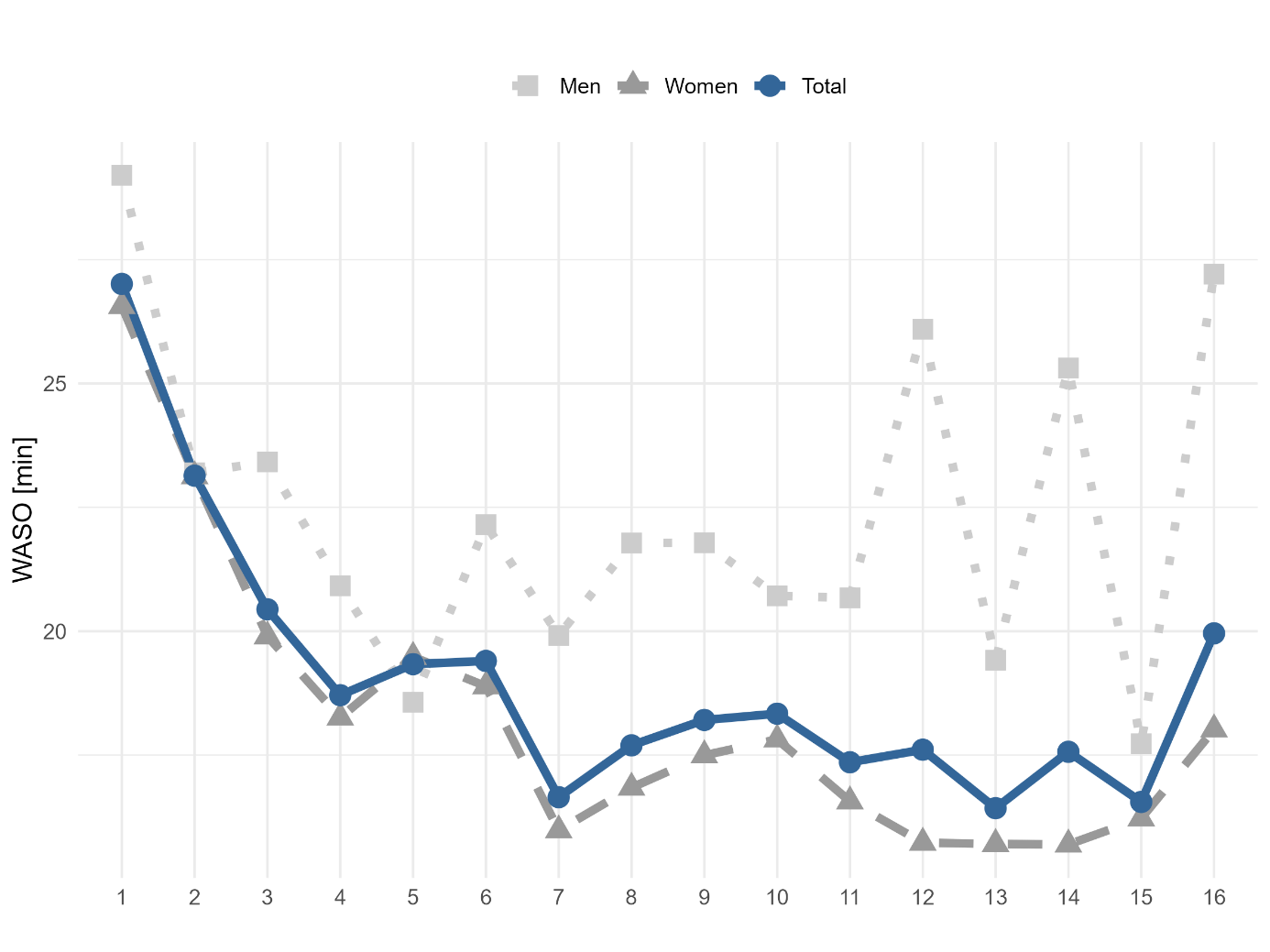


Figure S4. Course of the average sleep efficiency (SE) in minutes for the total group of the treatment group and separately for gender over the time between T0 and T1 per week.
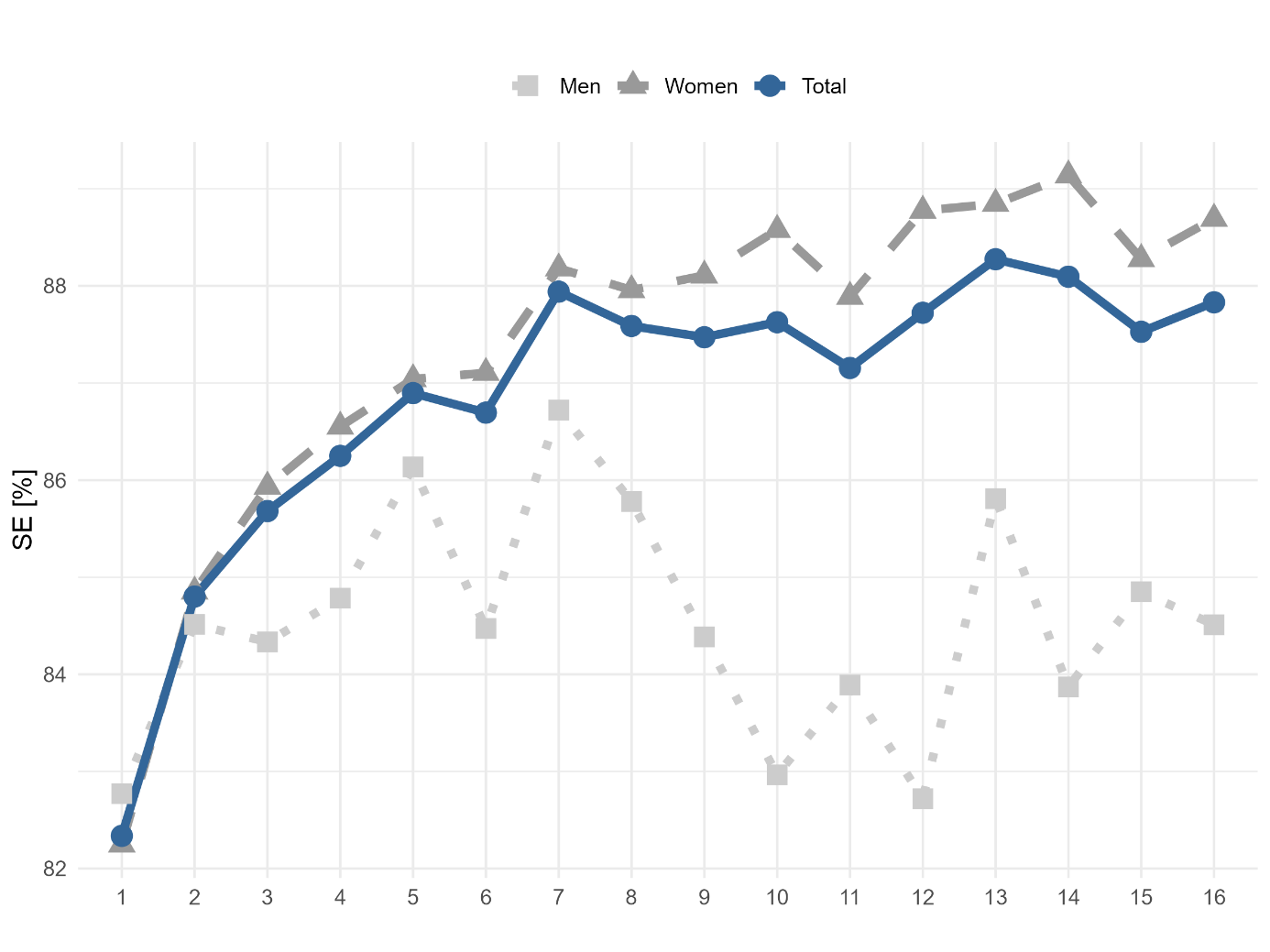


Figure S5. Course of the average sleep quality (SQ) for the total group of the treatment group and separately for gender over the time between T0 and T1 per week.
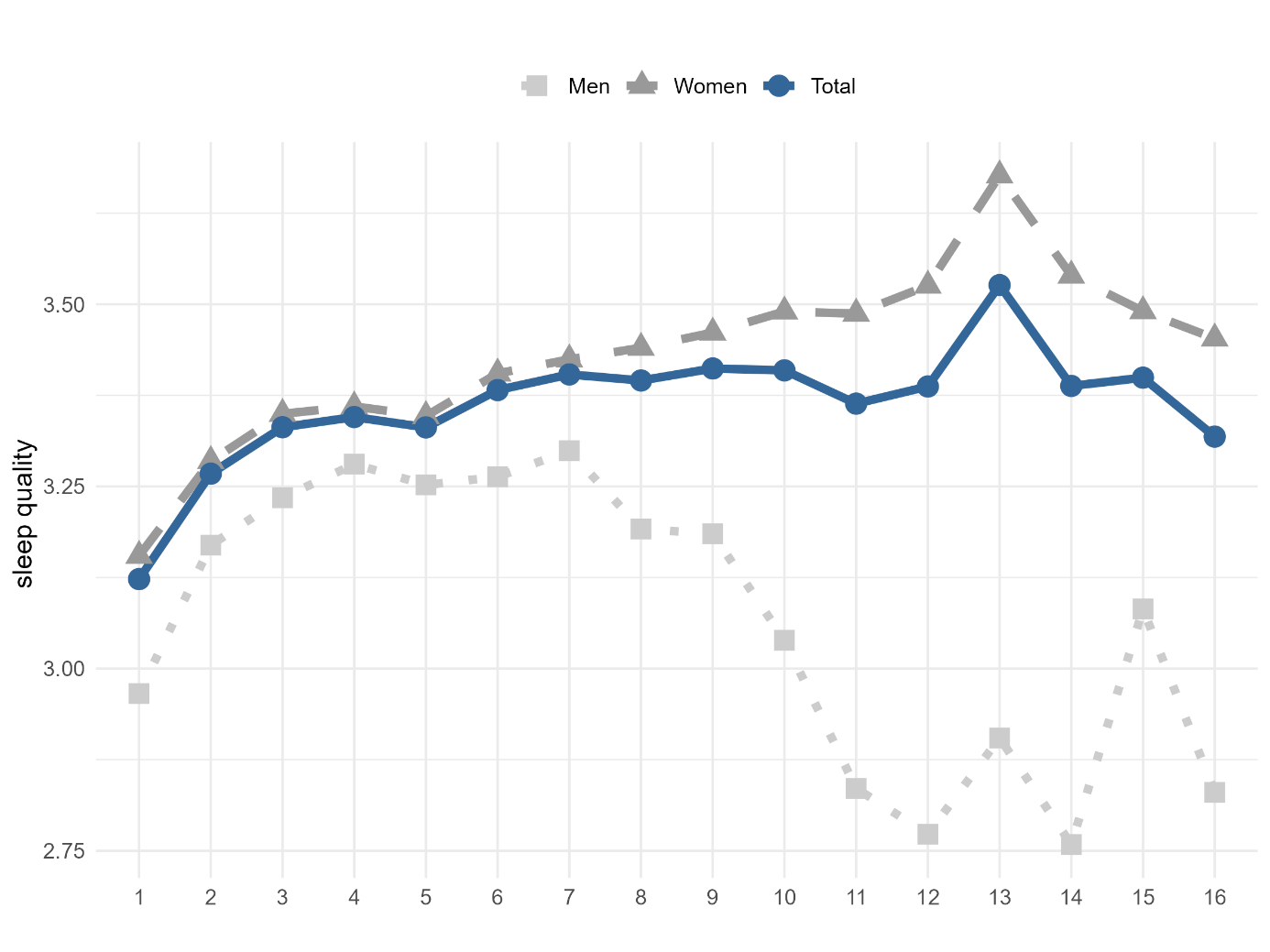


Table S8.

*Descriptive statistics (n, M, SD), and correlations of self-reported efficacy of sleep course, adherence, and treatment outcomes.*

| Variable | *M* | *SD* | Usefulness | Continued Mental Engagement | Continued Sleep Documentation | Sleep diary entries | Duration ΔT0-T1 |
| --- | --- | --- | --- | --- | --- | --- | --- |
| 1. Usefulness | 4.08 | 1.35 | ─ |  |  |  |  |
| 1. Continued Mental Engagement | 1.88 | 2.14 | 0.63*** | ─ |  |  |  |
| 1. Continued Sleep Documentation | 3.66 | 1.42 | 0.29*** | 0.36*** | ─ |  |  |
| 1. Sleep diary entries (No.) | 36.91 | 23.59 | 0.22** | 0.10 | 0.36*** | ─ |  |
| 1. Duration ΔT0-T1 | 69.04 | 15.83 | -0.18* | -0.15* | -0.06 | -0.03 | ─ |
| 1. ISI T0 | 12.97 | 3.17 | -0.08 | 0.02 | 0.15* | 0.09 | 0.17* |
| 1. SHI T0 | 30.05 | 5.07 | -0.19** | -0.10 | -0.04 | 0.05 | 0.16* |
| 1. Δ ISI T0-T1 | -4.79 | 3.72 | -0.23** | -0.09 | 0.01 | -0.21* | 0.03 |
| 1. Δ ISI T1-T2 | -0.91 | 3.46 | -0.33*** | -0.15* | -0.15* | -0.10 | 0.02 |
| 1. Δ SHI T0-T1 | -3.46 | 4.49 | -0.09 | -0.12 | -0.01 | -0.12 | 0.05 |
| 1. Δ SHI T1-T2 | -1.34 | 3.54 | -0.12 | 0.01 | -0.06 | -0.01 | 0.02 |

*Note*. *Usefullness* indicates subjective sleep improvement (item “The course has helped me to sleep better in the long term”).
